# Mobile Intracardiac Mass after Inguinal Hernia Repair: An Unresolved Treatment Dilemma

**DOI:** 10.1155/2015/375089

**Published:** 2015-08-16

**Authors:** Fahad Almehmadi, Mark Davis, Sheldon M. Singh

**Affiliations:** ^1^Schulich Heart Center, Sunnybrook Health Sciences Center, University of Toronto, 2075 Bayview Avenue, Toronto, ON, Canada M4N 3M5; ^2^King Saud Bin Abdulaziz University of Health Sciences, Jeddah, Saudi Arabia

## Abstract

Right heart thrombi (RHT) are rare but well-described entity in literature. Their isolation has been considered as confirmatory for the diagnosis of venous thromboembolism (VTE). Even though their isolation aids the diagnosis, physicians are faced with a difficult management dilemma giving the paucity of data to support any treatment decision. We present a case of RHT in an 81-year-old man who presented to hospital with a large mobile right heart thrombus in transit seen on transthoracic echocardiogram (TTE). He was successfully treated with anticoagulation alone. This case highlights the importance of TTE in establishing the diagnosis and describes the interplay of factors influencing treatment decision.

## 1. Introduction

Right heart thrombi (RHT) are rare manifestation of venous thromboembolism (VTE); their presence is considered a marker for higher clot burden and worse outcome [1–3]. Guidelines for management of VTE are well established [4]. But little is known about the optimum treatment strategy for RHT.

We present a case of right heart thrombi discovered on TTE emphasizing the utility of TTE in the setting of VTE. We aim also to describe the interplay of multiple clinical factors that may aid in treatment decision-making.

## 2. Case Presentation

**Figure 1:**
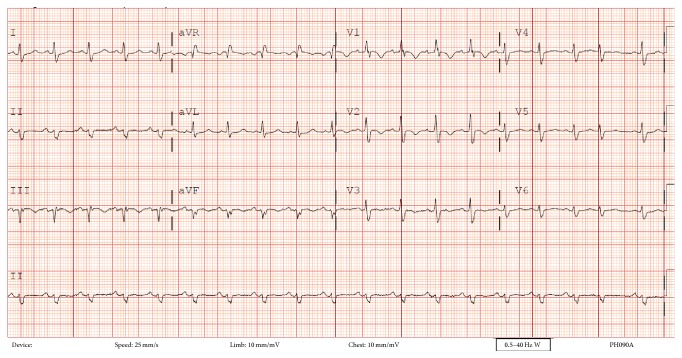
Electrocardiogram at presentation showing sinus tachycardia and S1Q3T3 pattern indicating right ventricular strain.

**Figure 2:**
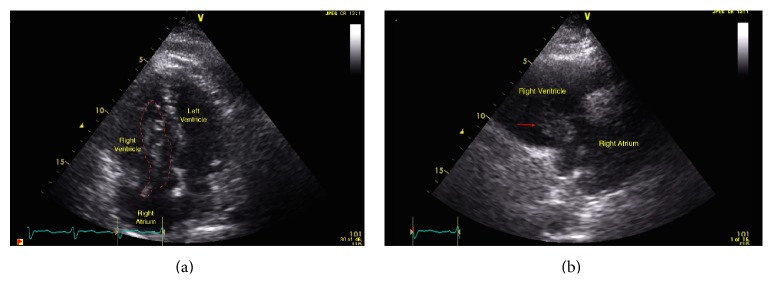
(a) An apical 4-chamber echocardiographic view of the heart showing the right heart thrombus protruding through the tricuspid valve. (b) A right ventricular inflow echocardiographic view of the heart showing the right heart thrombus protruding through the tricuspid valve.

An 81-year-old man presented with syncope 3 days after inguinal hernia repair. His past medical history was significant for a deep venous thrombosis that was diagnosed 2 months earlier. Electrocardiogram on presentation demonstrated sinus tachycardia with a S1Q3T3 pattern (Figure 1). Pulmonary embolism was suspected. Given a low creatinine clearance and a documented contrast allergy contrast CT angiography was not possible. As such, a transthoracic echocardiogram was performed demonstrating a dilated, hypokinetic right ventricle with preservation of apical contractile function (Supplemental Video in the Supplementary Material available online at http://dx.doi.org/10.1155/2015/375089) which has been reported to be consistent with a pulmonary embolism (McConnell's sign). Additionally a mobile serpiginous intracardiac mass straddling the tricuspid valve was present representing a thrombus in transit (Figures 2(a) and 2(b), Supplemental Video). Multiple bilateral large perfusion defects were observed in a ventilation perfusion scan confirming pulmonary emboli. Bilateral lower extremity deep venous thromboses were also present with leg Doppler evaluation. Fibrinolysis was contemplated given the presence of a large clot burden, right ventricular compromise, and presentation with syncope. However, given the increased bleeding risk due to age and renal dysfunction, conservative therapy with intense anticoagulation was initiated. During the course of therapy the patient experienced a brief episode of dyspnea and hypoxia presumed to be related to additional pulmonary embolism from the right heart thrombus given the fact that the thrombus visualized within the right heart initially was no longer present subsequent to this event. The patient remained hemodynamically stable with preserved oxygenation during his stay in the coronary care unit. He was subsequently discharged home.

## 3. Discussion

Right heart thrombi (RHT) can be one of many potential causes of a right heart mass including congenital structures (e.g., Chiari network, persistent Eustachian valve, and atrial septal aneurysm) and acquired causes (e.g., leads, vegetation, or tumors) [5, 6]. Clinical history and imaging will aid in diagnosis.

This case highlights the value of echocardiography with suspected pulmonary embolism. Although typical echocardiographic findings provide indirect evidence of pulmonary embolism, in the absence of prior cardiopulmonary disease these findings can be specific [7, 8]. Right heart thrombus (RHT) in transit has been reported in 4–10% of cases of pulmonary embolism [9]. This finding confirms the diagnosis, indicates a higher clot burden, worse pulmonary hemodynamics, and functional class, and is associated with a 10-fold higher mortality than that of isolated pulmonary embolism [1–3].

Treatment of RHT represents a management dilemma, given the absence of clear consensus treatment guidelines. Indeed, the need for appropriate therapy is most evident by the high mortality observed in the first 24 hours and an overall mortality in untreated patients approaching 100% [3, 5].

Management strategies of varying risk have been proposed including pharmacological therapy with either intensive anticoagulation or thrombolysis and invasive therapy with either a catheter-based or surgical embolectomy. Comparative effectiveness studies evaluating these strategies have been limited by their small sample size and lack of randomization, both of which do not allow for a true understanding of the risks and benefits of each approach. For example, Finlayson described 38 cases of right heart thrombi where a similar and high (20–62%) in-hospital mortality was present regardless of the treatment modality used [5]. In contrast, Ryu et al. suggested that thrombolysis may have a mortality benefit over other therapeutic approaches with a mortality rate reported at 11% compared to 23% and 28% observed in patients treated with surgical embolectomy and anticoagulation, respectively [3].

Thrombolytic therapy has been advocated as the first treatment modality [1, 3, 5, 10–13] due to its widespread availability in clinical practice and its ability to hasten thrombus breakdown thereby lowering the overall thrombus burden and improve right and left ventricular hemodynamics [5, 10]. Successful thrombolysis has been reported with both slow and rapid infusion of Tissue Plasminogen Activator (tPA) [14]. Thrombolysis, however, does have the limitation of increased risk of bleeding, repeat embolization with subsequent sudden death, and chronic thromboembolic pulmonary hypertension [12, 15]. Surgical embolectomy has been advocated as a rescue therapy [12, 15, 16]. Surgery has its inherent risks including the need to transport to a more experienced facility, anesthesia risk, and the inability to clear distal pulmonary circulation emboli [15, 17]. Despite this, surgery may be the only option in a critically ill patient [18–20].

Catheter aspiration thrombectomy has also been used with success in few reported cases [17, 21]. This modality may only be feasible in few highly specialized centers. Even when it is available, gaining a safe access to the right atrium can be very challenging in the setting of VTE.

Anticoagulation is not advocated as a sole therapy [1, 3, 5, 22]. However, as demonstrated in the case, other clinical factors may prevent patients from receiving more aggressive therapy. As such, this approach may be the only one available to patients.

Given the limitations of each available therapy and lack of consensus on the management of patients with RHT, a personalized approach accounting for patient and institutional factors must be adopted for each patient.

## Supplementary Material

Apical 4 chamber and right ventricular inflow views on transthoracic echocardiogram showing the mobile intracardiac thrombus protruding through the tricuspid valve.

## Abbreviations

RHT: Right heart thrombi
VTE: Venous thromboembolism.
